# Discovery of a Novel Activator of KCNQ1-KCNE1 K^+^ Channel Complexes

**DOI:** 10.1371/journal.pone.0004236

**Published:** 2009-01-21

**Authors:** Karen Mruk, William R. Kobertz

**Affiliations:** Department of Biochemistry and Molecular Pharmacology, Programs in Neuroscience and Chemical Biology, University of Massachusetts Medical School, Worcester, Massachusetts, United States of America; University of Virginia, United States of America

## Abstract

KCNQ1 voltage-gated K^+^ channels (Kv7.1) associate with the family of five KCNE peptides to form complexes with diverse gating properties and pharmacological sensitivities. The varied gating properties of the different KCNQ1-KCNE complexes enables the same K^+^ channel to function in both excitable and non excitable tissues. Small molecule activators would be valuable tools for dissecting the gating mechanisms of KCNQ1-KCNE complexes; however, there are very few known activators of KCNQ1 channels and most are ineffective on the physiologically relevant KCNQ1-KCNE complexes. Here we show that a simple boronic acid, phenylboronic acid (PBA), activates KCNQ1/KCNE1 complexes co-expressed in *Xenopus* oocytes at millimolar concentrations. PBA shifts the voltage sensitivity of KCNQ1 channel complexes to favor the open state at negative potentials. Analysis of different-sized charge carriers revealed that PBA also targets the permeation pathway of KCNQ1 channels. Activation by the boronic acid moiety has some specificity for the Kv7 family members (KCNQ1, KCNQ2/3, and KCNQ4) since PBA does not activate Shaker or hERG channels. Furthermore, the commercial availability of numerous PBA derivatives provides a large class of compounds to investigate the gating mechanisms of KCNQ1-KCNE complexes.

## Introduction

The five KCNQ voltage-gated K^+^ channels (Kv7) are responsible for membrane excitability, cardiac rhythmicity, and maintaining salt and water homeostasis [1]. The KCNQ family is divided by their tissue expression: KCNQ1 (Q1)^1^ channels are expressed throughout the body, but are noticeably absent from the central nervous system where KCNQ2–5 channels are primarily found [2]. KCNQ2–5 subunits form homo- and heterotetrameric K^+^ channels. KCNQ2/3 (Q2/Q3) contribute to the M-current and mutations in these channels cause benign familial neonatal convulsions (BFNC) [3]. Homotetrameric KCNQ4 (Q4) channels have also been implicated in disease. Mutations in Q4 cause an autosomal dominant form of progressive hearing loss in humans [4], [5]. In contrast, Q1 channels only form homotetramers and function in non-excitable as well as excitable tissues [6]. In order to properly function in these diverse tissues, Q1 channels co-assemble with KCNE peptides, affording complexes with different gating properties and pharmacological sensitivities [7]. Although KCNE peptides promiscuously assemble with many voltage-gated K^+^ channels in expression systems [7], the physiological relevance of most of the Q1-KCNE (E1, E2, and E3) complexes are well-established. Q1 subunits form a complex with KCNE1 (E1) peptides in the heart and inner ear, generating the cardiac I_Ks_ current and providing an avenue for K^+^ to enter the endolymph, respectively [8]–[10]. Mutations in either Q1 or E1 that decrease the conductance of the complex prolong the cardiac action potential [11], leaving individuals with these mutant proteins susceptible to long QT syndrome. In contrast to the slowly activating and deactivating Q1/E1 complex, both Q1/E2 and Q1/E3 complexes are constitutively conducting and contribute to K^+^ recycling in epithelial cells of the gastrointestinal tract [12], [13].

Although the different KCNE peptides have diametrically opposite effects on Q1 channel function, the molecular mechanisms involved in KCNE modulation of Q1 channel gating are just starting to be revealed [14]–[16]. Simple, small molecules that activate Q1-KCNE complexes would be valuable tools for investigating KCNE modulation of Q1 channel gating. Indeed, low-affinity blockers such as the quaternary ammoniums have been instrumental in the biophysical characterization of the permeation pathway of K^+^ channels [17]–[20]. However, small molecule activators of voltage-gated K^+^ channels are very rare [21] and often synthetically challenging to derivatize. Moreover, KCNE peptides are known to affect the sensitivity of pharmacological agents that modulate Q1 function [22]. Inhibitors of Q1 function are typically more potent when the channels are co-assembled with KCNE peptides [23]–[26]. Conversely, small molecules that activate homomeric Q1 channels are often ineffective on Q1/E1 complexes. Two known examples of this phenomenon are the Q1-specific activator, R-L3, and the recently discovered KCNQ activator, zinc pyrithione [21], [27], [28]. The non-specific Cl^−^ channel blockers, mefanamic acid and DIDS, are the exception to the rule as they cross-react with and activate Q1/E1 complexes [26], [29]. Thus, there remains a dearth of small molecule activators for the biophysical study of Q1-KCNE complexes.

During our initial efforts to chemically activate Q1 channels by specifically modifying the arginines in the voltage sensor, we serendipitously discovered that some boronates were modulators of Q1/E1 complexes. Examination of a small panel of boronic acids revealed that the aromatic derivative, phenylboronic acid (PBA), activates Q1/E1 complexes at millimolar concentrations. Activation of Q1/E1 by PBA is due to a shift in the voltage sensitivity of the complex and is specific for the boronic acid moiety. The permeation pathway is also affected by PBA since the magnitude of Q1 channel activation is dependent on the charge carrier. PBA shows some selectivity as it activates other members of the KCNQ family, but does not activate Shaker or hERG K^+^ channels. Since derivatives of PBA are common building blocks for organic synthesis, there currently exists a vast array of structurally diverse phenylboronic acids with varied physiochemical properties. The accessibility to thousands of PBA derivatives provides an opportunity to systematically dissect the mechanisms of Q1-KCNE gating and may lead to the discovery of a potent activator of Q1/E1 complexes for the treatment of cardiac arrhythmias.

## Materials and Methods

### Molecular Biology

cDNA encoding human KCNQ1, E1, and E3 were individually subcloned into the vector pSG01MX, which contains the 5′ and 3′UTRs from the *Xenopus* β-globin gene for increased protein expression. Inactivation-removed Shaker (Shaker-IR) was in pBluescriptII KS (+). h-KCNQ2, Q3, and Q4 were kindly provided by G. Seebohm and hERG was kindly provided by M. C. Sanguinetti. The constructs were linearized with the appropriate restriction enzyme (New England Biolabs) and cRNA was synthesized using *in vitro* run-off transcription with SP6 or T7 polymerase (Promega).

### Electrophysiology

Oocytes were surgically removed from *Xenopus laevis*. The extraction procedure and care of *Xenopus laevis* was approved by the University of Massachusetts Institutional Animal Care and Use Committee. Oocytes were defolliculated using 2 mg/mL collagenase (Worthington Biochemical Corp.) in OR2 solution containing (in mM): 82.5 NaCl, 2.5 KCl, 1 MgCl_2_, 5 HEPES, pH 7.4 for 60–80 minutes. Isolated oocytes were rinsed and stored in ND96 storage solution containing (in mM): 96 NaCl, 2 KCl, 1.8 CaCl_2_, 1 MgCl_2_, 5 HEPES, 50 µg/mL of both gentamicin and tetracycline pH 7.4, at 18°C. Oocytes were microinjected 24 h after surgery with Shaker, Q1, Q4, or hERG mRNA (15.2 ng). Additionally, Q1 mRNA (7.6 ng) was co-injected with E1 or E3 mRNA (3.8 ng). For Q2/Q3 heteromeric channels, an equal amount of mRNA (7.6 ng) for each subunit was injected. After 2–4 days, currents were recorded using Warner Instrument OC-725 two-electrode voltage clamp (TEVC), and the data were acquired with Digidata 1322A using pClamp 9 (Axon Instruments). Electrodes were filled with: 3 M KCl, 5 mM EGTA, 10 mM HEPES, pH 7.6. Currents were measured in ND96 recording buffer containing (in mM): 96 NaCl, 2 KOH, 0.3 CaCl_2_, 1 MgCl_2_, 10 HEPES, pH 7.6. All chemical compounds were from Sigma Aldrich and dissolved directly into ND96 recording buffer to a final concentration of 10 mM unless otherwise noted. For the initial Borax experiments, 10 mM sodium tetraborate was used as a buffer instead of HEPES. The time courses of current changes upon compound application and washout were generated by repeatedly depolarizing and measuring the change in current at the end of the pulse. Channels were held at −80 mV and pulsed to +40 mV (0 mV for hERG) for 2 s (100 ms for Shaker) every 30 s to illicit current. For EC_50_ experiments, current changes were measured for a range of PBA concentrations (1–10 mM). Current-voltage relationships were measured in the presence or absence of 10 mM PBA by holding at −80 mV and stepping to a series of test potentials for 4 s in 10 mV increments, followed by a tail pulse at −30 mV. For Q1/E3 complexes, the current-voltage relationships were measured in KD50 containing (in mM): 48 NaCl, 50 KOH, 0.3 CaCl_2_, 1 MgCl_2_, 10 HEPES, pH 7.6 by holding at −80 mV and pulsing to a series of potentials for 2 s in 20 mV increments, followed by a tail pulse to −80 mV. For charge carrier experiments, currents were measured in modified KD50 containing either 50 mM KOH, RbCl, or CsCl.

### Data Analysis

Analysis of data was performed with Clampfit 9 (Axon Instruments) and Prism 5 software (Graphpad). The maximal change in current upon PBA washout at 40 mV was calculated as Δg_max_. EC_50_ values were calculated by plotting the Δg_max_ values as a function of PBA concentration and fitting the data to a hyperbola. The amplitude of tail currents was measured 6 ms (100 ms for Q1) after repolarization to −30 mV (−80 mV for Q1/E3 and 50 mM external charge carriers) and normalized such that the maximal tail current in the absence of drug was equal to 1. Normalized tail currents were plotted versus the test potential to produce activation curves. Activation curves were fit to the Boltzmann equation: I_tail_ = A1+(A2−A1) / (1+e^((V−V^½^)*(−zF/RT))^), where V_½_ is the voltage of half-maximal activation and z is the slope factor. A Student's paired *t*-test was performed to determine whether PBA activation was significantly different than activation by benzyl alcohol. Deactivation time constants for Q1 were measured after a depolarizing pulse to 40 mV and fitting the tail current at −80 mV to a single exponential. Time constants were fit after recovery from inactivation. For the comparison of outward and inward currents, the amplitude of the Q1 current was measured 2 s after depolarization for outward, 100 ms after repolarization for inward. The outward and inward currents were normalized to 1 after the onset of PBA inhibition (defined as time = 0). Subsequent values were plotted as percent increase as a function of PBA exposure time.

## Results

**Figure 1:**
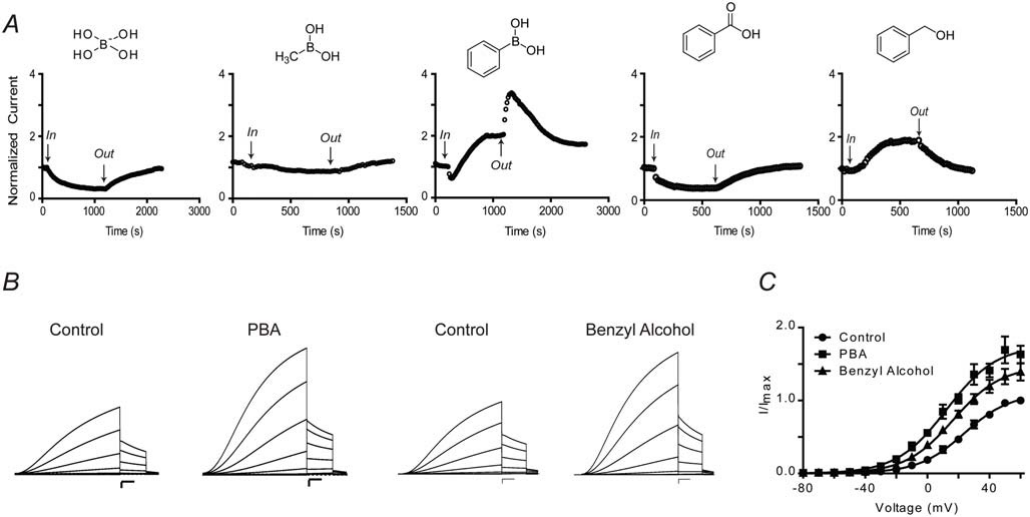
Modulation of Q1/E1 channels by boronates. (A) Time course of Q1/E1 current measured in ND96 at +40 mV at the end of a 2 s pulse. The current was normalized before compound application. Borax reversibly inhibits while methylboronic acid has little to no effect on channel current. Phenylboronic acid (PBA) initially inhibits current and then slowly potentiates. Benzoic acid reversibly reduces Q1/E1 channel current whereas benzyl alcohol reversibly activates the channel complex. (B) Families of currents recorded before and during treatment with PBA or benzyl alcohol. Currents were elicited by 4 s step test potentials from −80 to +60 mV in 10 mV increments from a holding potential of −80 mV followed by a tail pulse to −30 mV. Dashed line indicates zero current. Scale bars represent 1 µA and 0.5 s. (C) Voltage-activation curves for Q1/E1 calculated from tail current analysis. Solid curves represent Boltzmann fits to the data. Data are presented as the mean±SEM (n = 10).

Borax is a commonly used buffer for protein modification reactions that specifically neutralizes positively charged arginine residues [30]; thus, we initially determined whether borate buffer would have any effect on Q1/E1 complexes. Figure 1A shows normalized Q1/E1 current elicited by 40 mV, 2-s depolarizations in standard ND96 buffer. Switching the solution to a buffer that contains 10 mM sodium tetraborate resulted in reversible inhibition of the Q1/E1 complex. Intrigued by the inhibitory effect of borate, we determined whether boronic acid derivatives had a similar effect on Q1/E1 complexes. Methylboronic acid in ND96 with HEPES as the buffer had little to no effect on Q1/E1 complex function; however, perfusion of 10 mM phenylboronic acid (PBA) caused a rapid inhibition followed by a slower activation, resulting in a net doubling of current (Figure 1A). Upon washout of PBA, inhibition was quickly and completely relieved, resulting in a dramatic rise in current amplitude. Although PBA's inhibitory effect was rapidly reversible, activation of Q1/E1 complexes slowly diminished upon removal of PBA, but never fully washed out. To determine whether activation was due to a shift in the voltage sensitivity of Q1/E1 complexes, we measured the effect of PBA at different voltages (Figure 1B). Tail current analysis (Figure 1C) in the presence PBA resulted in a left-shift of the midpoint of activation (V_1/2_) and a decrease in the voltage-dependence (z) of Q1/E1 complexes (Table 1). Since PBA both activates and inhibits Q1/E1 complexes, we utilized the kinetic difference in washout to measure the maximal activation of Q1/E1 complexes by PBA. Upon washout of PBA, inhibition was quickly relieved resulting in a rapid rise in current (Figure 1A) and the maximum current was measured and defined as Δg_max_ (Table 1). Making this measurement with different concentrations of PBA afforded an EC_50_ of 1.6 mM for Q1/E1 complexes.

**Table 1:** Electrophysiological Properties of KCNQ Channels in the presence of PBA or Benzyl Alcohol.^a^

	Control		Phenylboronic Acid (PBA)					Benzyl Alcohol	
	V_1/2_ (mV)	*z*	Δ V_1/2_ (mV)	Δ *z*	Δg_max_	EC_50_ (mM)	Δ V_1/2_ (mV)	Δ *z*
**Q1**	−31.8±1.6	2.39±0.06	−13.8±1.6*	0.51±0.11	1.5±0.1	0.10±0.02	−4.6±5.5	0.51±0.18
**Q1/E1**	23.4±1.2	1.70±0.04	−12.6±2.7*	−0.23±0.07	3.0±0.1	1.6±0.5	−4.4±2.4	−0.23±0.07
**Q1/E3**	26.6±3.0	0.47±0.02	3.3±5.4*	0.33±0.04	3.9±0.2	6.1±5.7	2.7±4.9	0.35±0.06
**Q2/Q3**	−44.6±1.4	3.08±0.11	−9.8±2.3*	−0.32±0.34	3.0±0.3	3.7±1.4	−5.9±2.9	0.31±0.17
**Q4**	−15.6±1.1	2.45±0.06	−13.7±1.3*	−0.44±0.11	>10	>10	−0.8±2.9	−0.53±0.08

a Data from individual activation curves obtained from 4–10 oocytes. Activation curves were fit to a Boltzmann function. V_½_ is the voltage of half-maximal activation and *z* is the slope factor. ΔV_1/2_ and Δ *z* are the changes induced by addition of 10 mM compound. Δg_max_ values were determined during the washout of inhibition, as described in the Materials and Methods. EC_50_ values were determined during the washout of inhibition for PBA concentrations ranging from 1–10 mM. All values are mean±SEM except for EC_50_ values, which are reported as the error of the fit to a hyperbola.

* Indicates significant (Student *t*-test; p<0.05) when compared to benzyl alcohol.

To examine the importance of the boronic acid moiety, we tested the structurally similar compounds: benzoic acid and benzyl alcohol. Perfusion of benzoic acid reversibly inhibited Q1/E1 whereas benzyl alcohol reversibly activated the complex, but to a lesser extent than PBA (Figure 1). Benzyl alcohol also had a significantly smaller effect on the V_1/2_ of the complex compared to PBA (Figure 1C and Table 1). Therefore, we subsequently compared PBA and benzyl alcohol to determine whether the geminal diol of the boronic acid would activate other K^+^ channels and Q1-KCNE complexes.

**Figure 2:**
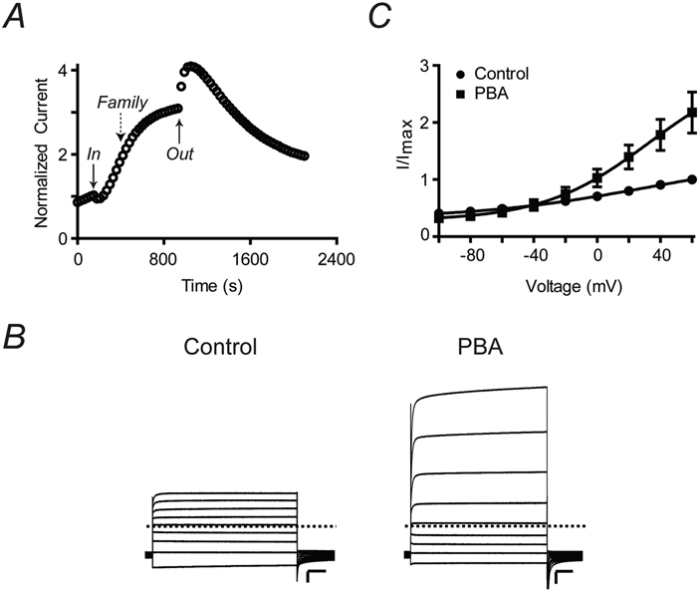
PBA activates Q1/E3 complexes. (A) Time course of Q1/E3 current measured in ND96 at +40 mV at the end of a 2 s pulse. The current was normalized before PBA application. PBA initially inhibits then slowly potentiates channel current. (B) Families of currents recorded in high external potassium (50 mM) before and during treatment with PBA. Currents were elicited by 2 s test potentials from −100 to +60 mV in 20 mV increments. The holding and tail potentials were −80 mV. Dashed line indicates zero current. Scale bars represent 1 µA and 0.5 s. (C) Voltage-activation curves for Q1/E3 calculated from tail current analysis. Solid curves represent Boltzmann fits to the data. Data are presented as the mean±SEM (n = 10).

Q1 channels co-expressed with KCNE3 (E3) were also activated by PBA (Figure 2A). In contrast to Q1/E1, Q1/E3 currents were primarily activated by PBA though a small amount of recovery from inhibition was observed as a rapid increase in current when the reagent was washed out. Since Q1/E3 complexes are open at negative potentials, we used a high external potassium solution (50 mM) to visualize both the outward and inward currents generated from a family of test potentials (Figure 2B). Although these conditions enabled us to measure the inward currents, Q1/E3-expressing oocytes became unstable during the long time course needed to observe complete activation by PBA (1000 s). Therefore, we measured the current-voltage relationships after 500 s of PBA application (dashed arrow in Figure 2A). Tail current analysis revealed that PBA activation of Q1/E3 complexes occurred only at potentials greater than −40 mV (Figure 2C). Accordingly, PBA did not shift the V_1/2_ of the complex, but apparently increased the voltage dependence of the Q1/E3 complex (Table 1). This effect was not specific for the boronic acid moiety because benzyl alcohol activated Q1/E3 similarly.

**Figure 3:**
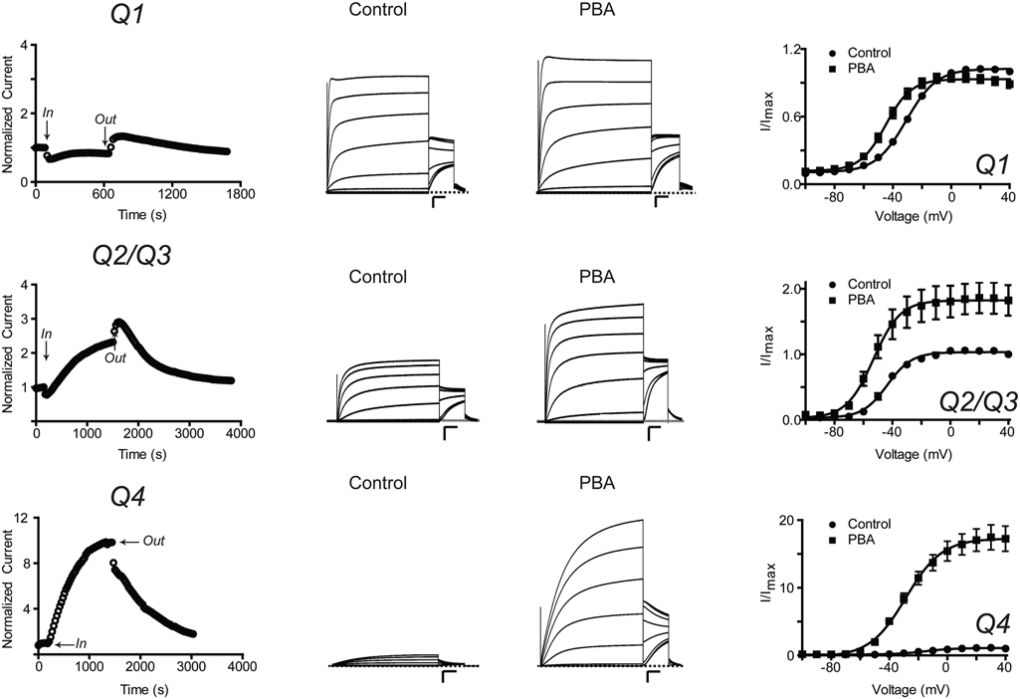
PBA activates all tested members of the KCNQ family. *Left panel:* Time courses of current recorded in ND96 at +40 mV at the end of a 2 s pulse. The current was normalized before PBA application. PBA initially inhibits and then slowly potentiates Q1 and Q2/Q3 current. PBA only activates Q4 channels. *Middle panel:* Families of currents recorded before and during treatment with PBA. Currents were elicited by 4 s test potentials from −100 to +60 mV in 10 mV increments from a holding potential of −80 mV followed by a tail pulse to −30 mV. Dashed line indicates zero current. Scale bars represent 1 µA and 0.5 s. *Right panel:* Voltage-activation curves calculated from tail current analysis. Solid curves represent Boltzmann fits to the data. Data are presented as the mean±SEM (n = 4–6).

**Figure 4:**
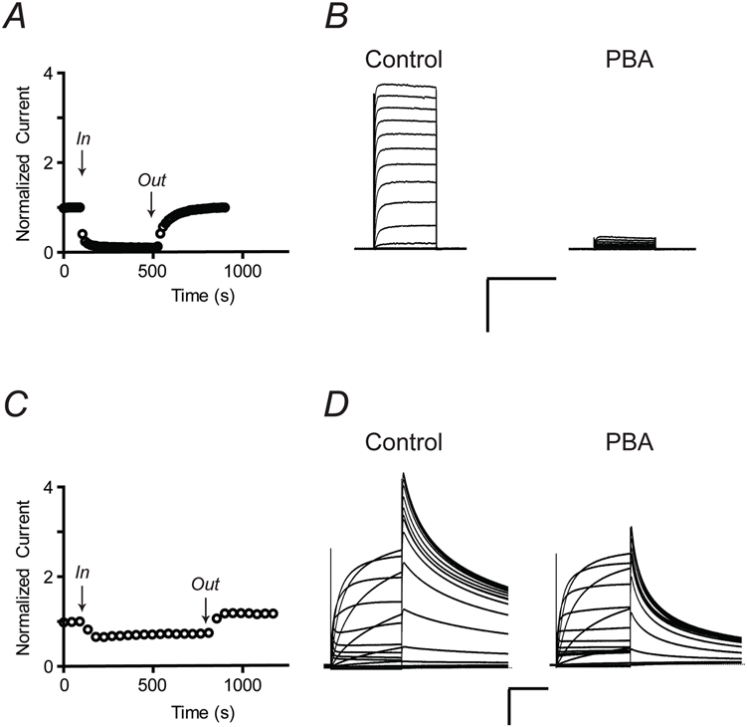
PBA inhibits other Kv channels. (*A*) Time course of Shaker (inactivation removed) current measured in ND96 at +40 mV, 100 ms pulse when 10 mM PBA was applied through the bath solution. (*B*) Families of Shaker currents recorded before and during treatment with PBA. Currents were elicited by 100 ms step test potentials from −100 to +60 mV in 10 mV increments from a holding potential of −80 mV. Dashed line indicates zero current. Scale bars represent 1 µA and 0.1 s. (*C*) Time course of hERG current measured in ND96 at 0 mV, 2 s pulse when 10 mM PBA was applied through the bath solution. (*D*) Families of hERG currents recorded before and during treatment with PBA. Currents were elicited by 2 s step test potentials from −100 to +60 mV in 10 mV increments from a holding potential of −80 mV. Dashed line indicates zero current. Scale bars represent 1 µA and 0.5 s.

Since Q1-KCNE complexes were activated by PBA, we next asked whether activation was KCNE-specific by examining other members of the KCNQ family. Specifically, we examined the physiologically relevant heterotetrameric Q2/Q3 channels and homotetrameric Q4 channels. For homomeric Q1 channels, activation by PBA was offset by inhibition, resulting in a negligible increase in current. However, PBA significantly left-shifted the V_1/2_ of the channel and this activation was observed during washout (Figure 3, Q1 panel). The overall effect of PBA on Q2/Q3 heterotetrameric channels was activation (Figure 3, Q2/Q3 panel). Benzyl alcohol also activated Q2/Q3 channels, however, to significantly lesser extent (Table 1). Strikingly, Q4 channels were *only activated* by PBA (Figure 3, Q4 panel). A ∼10-fold increase in current was observed at 40 mV and the V_1/2_ of the complex was significantly left-shifted (Table 1). Moreover, activation is specific for the boronic acid moiety since benzyl alcohol did not shift the V_1/2_ (Table 1). To determine whether PBA activation was specific for KCNQ family members, we examined two different voltage-gated K^+^ channels: Shaker and hERG. Both the inactivation removed variant of Shaker (Shaker-IR) and hERG were only reversibly inhibited by 10 mM PBA (Figure 4). Therefore, activation by PBA appears to be somewhat specific for KCNQ1-KCNE channel complexes and KCNQ channels.

**Figure 5:**
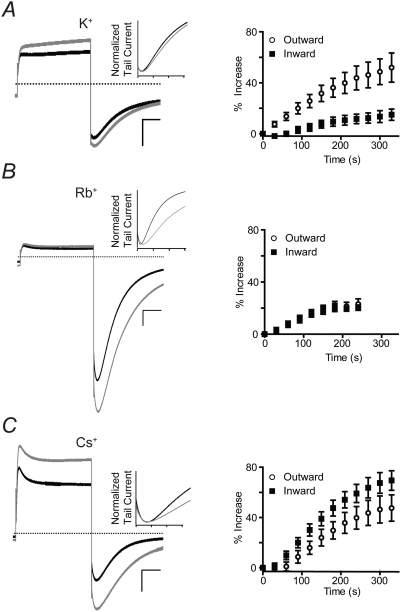
Activation of Q1 by PBA is dependent on the external charge carrier ion. Current traces were recorded in (A) 50 mM K^+^, (B) Rb^+^, or (C) Cs^+^. *Left panel:* Representative overlaid traces elicited by a +40 mV test and −80 mV tail pulse before and after the onset of PBA potentiation. *Inset:* Normalized tail currents comparing the deactivation kinetics before and after the onset of PBA potentiation. Tick marks represent 200 ms. *Right panel:* Time course of current recorded during the PBA potentiation phase. Outward current measured at the end of a 2 s pulse to +40 mV; maximal inward current measured during the −80 mV tail pulse. Time zero is the amount of current after initial inhibition but before potentiation by PBA. Data are represented as the mean±SEM (n = 5–10).

Because Q1 channels rapidly flicker between open and closed, single channel events cannot be directly observed; therefore, we indirectly determined whether PBA alters the permeation pathway of Q1 channels during the potentiation phase. We examined PBA potentiation in the presence of external cations with diameters larger than K^+^ since the Q1 channel pore readily conducts both Rb^+^ and Cs^+^ [31], [32]. Oocytes expressing Q1 channels were initially bathed in 50 mM external K^+^, Rb^+^, or Cs^+^. PBA (10 mM) was added and after the onset of inhibition, the cell was depolarized to 40 mV and returned to −80 mV to elicit outward and inward currents, respectively (Figure 5, black traces). The outward and inward currents were normalized and defined the current level at time = 0. During the potentiation phase, outward and inward currents were measured every 30 s until equilibrium was reached (Figure 5, right graphs); the final trace is shown in gray in the left panel of Figure 5. For K^+^, PBA caused a greater increase in outward than inward current (Figure 5A) while in Rb^+^, both outward and inward current increased equally (Figure 5B). In contrast, PBA caused a greater increase in inward than outward currents when measured in Cs^+^ (Figure 5C). The deactivation kinetics during the potentiation phase (Figure 5, insets) with the different charge carriers were also measured. PBA slows Q1 channel closing when the larger Rb^+^ or Cs^+^ ions are charge carriers whereas PBA has no significant effect on closing kinetics with external K^+^ (Table 2). Since permeation and voltage-dependent gating are intrinsically coupled in Q1 channels [32], we also measured the PBA-induced changes in voltage sensitivity with the different charge carriers (Table 3). PBA caused a −14 mV shift in the V_1/2_ of Q1 channels in both ND96 (2 mM K^+^_ext_) and 50 mM K^+^_ext_ (Table 1 and 3). However, in the presence of the larger Rb^+^ and Cs^+^ ions, PBA left-shifted the V_1/2_ an additional 6 mV. In total, these results show that activation of Q1 by PBA is dependent on the external charge carrier.

**Table 2:** Deactivation rates of Q1 with different charge carriers.^b^

	Deactivation τ (ms)		
	K^+^	Rb^+^	Cs^+^
**Before PBA**	360±30	480±30	300±20
**PBA (inhibition)**	510±30	710±90	430±30
**PBA (activation)**	630±60	1240±200*	650±60*

b Rates of deactivation were measured by fitting the tail currents to a single exponential after recovery from inactivation. Data is represented as the mean±SEM (n = 5–10).

* Indicates significant (Student *t*-test; p<0.05) when compared to inhibition by PBA.

**Table 3:** Electrophysiological Properties of Q1 with different charge carriers.^c^

	Control		PBA	
	V_1/2_ (mV)	z	Δ V_1/2_ (mV)	Δ z
**K+**	−26.8±2.4	1.6±0.1	−14.3±3.1	0.02±0.30
**Rb+**	−26.5±1.2	1.7±0.1	−21.6±1.7*	0.6±0.20*
**Cs+**	−30.8±1.4	2.2±0.1	−19.3±1.7*	0.4±0.20*

c Data from individual activation curves obtained from 5–10 oocytes. Activation curves were fit to a Boltzmann function. V_½_ is the voltage of half-maximal activation and *z* is the slope factor. ΔV_1/2_ and Δ *z* are the changes induced by addition of 10 mM PBA. All values are mean±SEM.

* Indicates significant (Student *t*-test; p<0.05) when compared to K^+^.

## Discussion

Motivated by the fortuitous discovery that Borax modulates Q1/E1 complexes, we determined whether the boronic acid moiety was uniquely responsible for modulation of KCNQ channels and KCNQ1-KCNE complexes. By examining structurally different boronic acids, we found that the aromatic derivative, PBA, activates Q1/E1 complexes at millimolar concentrations. PBA activation of Q1/E1 is specific for the boronic acid functional group because other similar aromatic derivatives (benzyl alcohol and benzoic acid) are significantly less effective or inhibitory. In contrast, both PBA and benzyl alcohol similarly activate the Q1/E3 complexes. Activation of the constitutively conducting Q1/E3 complex by PBA is only observed at voltages greater than −40 mV, which ostensibly increases the voltage-dependence of the complex. Homomeric Q1 channels are also modulated by PBA. At positive potentials, PBA inhibits and activates Q1 channels equally, resulting in no net change in current magnitude. Although PBA does not significantly increase the total current, the voltage sensitivity of Q1 channels is shifted with PBA such that they are open at more negative potentials. These results suggest that the presence of the KCNE peptides is not required for PBA to modulate the ion conducting subunit; however, an overall increase in current magnitude is only observed when Q1 channels co-assemble with KCNE peptides. Thus, PBA is a more effective activator of Q1-KCNE complexes.

In addition to Q1 channels, PBA activates the other members of the KCNQ family. Activation appears to be somewhat specific for KCNQ channels, as Shaker and hERG are not activated by PBA. Comparing activation curve data for the KCNQ family indicated that PBA activates these channels by shifting the midpoint of activation (V_1/2_). The maximal increase of current (Δg_max_) for KCNQ channels at 40 mV is: Q4≫Q1/E1≈Q2/Q3>Q1. This activation trend inversely correlates with the reported open probabilities for KCNQ channels [33]–[36], suggesting a rationale for the varied effectiveness of PBA. Testing this hypothesis, however, is hampered by the flickery nature of Q1/E1 complexes, which precludes the accurate measurement of the open probability of these complexes by either single channel recordings or noise analysis [37], [38].

Without the ability to perform traditional single channel analysis, we turned to Rb^+^ and Cs^+^ as charge carriers to indirectly examine the effect of PBA on the Q1 permeation pathway. We found that the inward and outward Q1 currents were differentially potentiated by PBA. PBA increased Q1 outward currents (compared to inward) when K^+^ was in the external bath and inward currents when Cs^+^ was present. The asymmetry of PBA activation of Q1 currents cannot be explained by increasing the number of channels and thus points to modulation of the ion conducting subunits. Consistent with this premise was the charge carrier dependence of PBA potentiation: the larger the diameter of the permeant ion, the more PBA increased the inward current (Figure 5). This result suggests that PBA alters the Q1 selectivity filter such that larger ions are more permeant. The larger Rb^+^ and Cs^+^ ions also enhanced PBA's effect on the V_1/2_ and deactivation kinetics of Q1 channels compared to K^+^. Although the sum of these results do not conclusively rule out an indirect modulatory mechanism, the data in total strongly favor PBA directly interacting with the KCNQ pore forming subunit.

One commonality of PBA modulation of KCNQ channels is the slow onset of potentiation (minutes). If PBA is directly binding to the channel, the slowed kinetics could be explained by a cytoplasmic or buried binding site. PBA binding may in fact be covalent, because boronic acids are known to form covalent, yet reversible bonds with diols [39], [40]. A covalent interaction is consistent with the previous observations that chemical modifications activate KCNQ channels [14], [15], [35], [41]. In addition, a slowly reversible covalent interaction may also explain the lack of complete washout with some KCNQ channels (Figures 1A, 2A and 3). Nonetheless, PBA may still be acting indirectly, activating signaling pathways in the cell. However, these indirect pathways must be specific for KCNQ channels since PBA activation was not observed for Shaker and hERG channels.

While PBA activates KCNQ channels, it does inhibit Kv channels weakly at 10 mM. Rapid inhibition was initially observed for all Kv channels, except Q4. The trend for inhibition is: Shaker>Q1≈hERG≈Q1/E1>Q2/Q3≈Q1/E3. hERG inhibition is a concern for small molecule drug design; however, PBA inhibition of hERG does not appear to occur by classic inner vestibule block [42]. First, PBA is not a positively charged molecule, which is typical for hERG blockers that bind to the hydrophobic residues in the S6 helix [43]. Second, inhibition is not specific for hERG, as the other Kv channels are similarly inhibited at this high concentration of PBA. Given that PBA inhibition is already weaker than activation for KCNQ channels, it should be possible to design second generation boronic acid activators that do not inhibit hERG and other Kv channels.

Unlike recently described activators of KCNQ channels, which are rendered less effective when co-assembled with KCNE peptides [27], [28], PBA activates Q1-KCNE complexes more effectively than homotetrameric Q1 channels. Both of these previous studies suggest that co-assembly with KCNE peptides prevents R-L3 and zinc pyrithione from binding to the Q1 channel subunit [21], [27], [28]. Since PBA activates Q1-KCNE complexes, future structure-function studies with PBA and other boronic acids should provide insight into its binding site and yield new tools to investigate the molecular mechanisms of Q1-KCNE gating. Although the potency of this unoptimized, simple molecule is modest, the non-toxicity of boronic acids (Borax), the catalogues full of boronic acids derivatives, and their synthetic utility makes PBA a potential pharmacophore for building potent activators of Q1/E1 complexes. Moreover, since boronic acid-bearing compounds are used clinically [44], their inclusion into small molecule libraries would generate an array of potential KCNQ activators.
